# Primary outcome measure use in back pain trials may need radical reassessment

**DOI:** 10.1186/s12891-015-0534-1

**Published:** 2015-04-14

**Authors:** Robert Froud, David Ellard, Shilpa Patel, Sandra Eldridge, Martin Underwood

**Affiliations:** Clinical Trials Unit, Warwick Medical School, University of Warwick, Gibbet Hill Road, Coventry, CV4 7AL UK; Norges Helsehøyskole, Campus Kristiania, Prinsens Gate 7-9, Oslo, 0152 Norway; Queen Mary University of London, 58 Turner Street, London, E1 2AB UK

**Keywords:** Low back pain, Outcome assessment, Measurement, Qualitative research, Roland morris disability questionnaire, Global transition question, Inclusive thinking

## Abstract

**Background:**

The answers to patient reported outcome measures and global transition questions for back pain can be discordant. For example, the most commonly used outcome measure in back pain trials, the Roland Morris Disability Questionnaire (RMDQ), can show improvement even though participants say that their back pain is worse. This gives cause for concern as transition questions are used as anchors to estimate minimally important change (MIC) thresholds on patient reported outcome measures such as the RMDQ. We aimed to explore and compare what people with back pain think when they respond to a transition question and when they complete the RMDQ.

**Methods:**

We purposively sampled people enrolled on a back pain randomised controlled trial who completed the RMDQ and two transition questions. One enquired about change in ability to perform tasks, the other about change in back pain. We sampled participants with discordance (in both directions), and participants with concordant scores. We explored participants’ thought processes using in-depth interviews.

**Results:**

We completed 35 in-depth interviews. People with discordant RMDQ change and transition question responses attend to different factors when responding to transition questions compared to people with concordant scores. In particular, those for whom the RMDQ change indicated greater improvement than transition questions, prioritised their pain ahead of functional disability. When completing the RMDQ, participants’ thought processes were comparatively more objective, and specific to each statement.

**Conclusion:**

Approaches to primary outcome assessment in back pain needs re-assessment. The RMDQ may be unsuitable for use as a primary outcome measure since patients may not attend to thinking about their back pain when completing it: patients’ abilities to cope with tasks can be independent of the change in their back pain. Some participants who improve on the RMDQ consider themselves globally worse. As transition questions can be driven by pain and other physical factors, transition questions should not be used to anchor minimally important change thresholds on the RMDQ.

**Electronic supplementary material:**

The online version of this article (doi:10.1186/s12891-015-0534-1) contains supplementary material, which is available to authorized users.

## Background

Low back pain (LBP) is a common and costly health complaint; its life-time prevalence may be as high as 84% [1]. Around 4% of the UK population take time off work because of low back pain, which equates to around 90 million working days lost and between eight and 12 million GP consultations per year [2,3]. Globally, LBP ranked number one for contributions to Years Lived with Disability (YLDs) in 2012 [4].

It is recommended that the performance of Patient Reported Outcome Measures (PROMs) for measuring health outcomes is carefully and systematically evaluated prior to their use in clinical trials [5]. Steps of this process can necessitate the use of a global transition question (TQ). A TQ is also a PROM but a special case, as in contrast to most instruments that cover one or more domains with multiple questions, TQs contain only a single question asking if a patient has improved or deteriorated since beginning treatment [6]. TQs facilitate evaluation of responsiveness and minimal important change (MIC) thresholds for PROMs, through being used as ‘anchors’ – that is to say, the PROM scores of those responding in a particular category of the TQ are categorised for analysis, and then used to dichotomise PROM score improvements; in Receiver Operator Characteristic (ROC) curves for example [7]. Well-performing PROMs are selected for use as outcome measures in clinical trials of treatments for chronic conditions, as well as for use in clinical practice [5]. In clinical practice, management decisions about individual patients can be based upon clinical assessments/examinations, biometrics, clinimetrics, and/or psychometrics. However, when evaluating changes in chronic conditions with variable courses (such as low back pain), for which there are no reliable objective tests, practitioners may simply ask patients whether or not they are any better—essentially the same question posed by a TQ. TQ responses have been shown to be discordant with low back pain (LBP) PROM scores and one criticism of TQs is that they may not always adequately measure change even though that is what they are designed to do [8,9].

Interventions for treating LBP are typically evaluated and compared using pragmatic Randomised Controlled Trials (RCTs), in which PROMs are typically used to evaluate participants’ health change and to explore between-group differences in health changes. The Roland Morris Disability Questionnaire (RMDQ) is the most commonly cited primary outcome measure in LBP trials [10]. In one of the largest trials of a physical therapy for LBP, participants’ RMDQ scores indicated an improvement on average, even in those participants who said that their back pain was worse [8]. In order to further assess whether it is appropriate to use the TQ to make inferences about the RMDQ (and *vice versa*), we used in-depth interviews to explore what people with back pain think about when they complete the RMDQ and when they respond to a TQ.

## Methods

In this study, participants were recruited from a sample of participants in a pilot cluster RCT (ISRCTN46035546) of informed shared decision-making. Participants, who were recruited to the trial from a National Health Service (NHS) physiotherapy department in Coventry UK, were aged 18 or over and due to receive physical therapy as a treatment for their non-specific LBP [11]. All participants needed to be fluent in English. The trial protocol and results are described in full detail elsewhere. Participants were informed by a trial participant information sheet that once they had returned their four-month follow-up questionnaire they may be approached to take part in a related interview study looking at how changes in back pain are measured and that further participation in this study would be voluntary [11,12]. We contacted participants by post, including a participant information sheet for the interview study and a form on which to register their interest. We obtained written informed consent at the time of the interview, directly from participants. After the interview, participants were given £20 (GBP) of high-street vouchers to thank them for their time. The study protocol received ethics approval from the National Research Ethics Service (NRES) Committee South East Coast – Brighton and Sussex Research Ethics Committee (REC) (11/LO/1190).

Participants in the trial had completed the original (1982) version of the RMDQ and TQs at baseline and at four months [6,13]. To explore the effect of TQ wording in this trial sub-study, at the design-stage of the trial we included two different TQs. One TQ asked ‘Since beginning treatment how would you describe the change in your low back pain?’ and the other asked ‘Since beginning treatment how would you describe the change in your ability to perform daily tasks?’ Each TQ utilised the same 7-point response options, where anchors ranged from 1=Completely better, to 7=Vastly worse (Footnote to Table 1).

**Table 1:** **Transition question responses**

**ID**	**Age**	**Ethnicity**	**Gender**	**Employment**	**Leg pain**	**RMDQ**	**RMDQ**	**RMDQ**	**TQ LBP**	**TQ**	**Discordance**
						**baseline**	**follow-up**	**change**		**daily tasks**	
1	63	White British	Female	Retired	No	15	12	-3	4	4	– –
2	70	White British	Female	Retired	Yes	11	13	2	3	3	++
3	57	White British	Female	Full Time	No	17	16	-1	3	3	00
4	61	White British	Female	Part Time	No	14	2	-12	3	3	– –
5	45	White British	Female	Full Time	Yes	10	4	-6	3	3	– –
6	47	White British	Female	Part Time	No	11	7	-4	3	4	0-
7	23	Asian British ^∗^	Female	Full Time	Yes	11	12	1	4	4	00
8	74	White British	Female	Retired	No	12	11	-1	3	3	00
9	49	Asian British ^∗^	Female	Full Time	No	18	8	-10	3	2	-0
10	57	White British	Female	Part Time	Yes	12	10	-2	3	3	00
11	58	White British	Female	Unassigned	No	12	10	-2	3	3	00
12	64	White British	Female	Unassigned	Yes	18	6	-12	3	2	-0
13	58	White British	Male	Not working	Yes	19	19	0	ND	ND	ND
14	54	White British	Male	Full Time	No	6	2	-4	2	2	++
15	55	White British	Male	Retired	Yes	14	0	-14	2	2	00
16	67	White British	Female	Retired	Yes	8	7	-1	3	2	0+
17	57	White British	Female	Full Time	Yes	7	16	9	7	7	00
18	56	White British	Female	Part Time	Yes	11	4	-7	2	2	00
19	73	White British	Female	Retired	No	10	10	0	4	4	00
20	64	White British	Female	Retired	Yes	15	16	1	4	4	++
21	65	White British	Female	Retired	Yes	4	2	-2	4	4	– –
22	34	White British	Female	Not working	No	4	0	-4	2	2	00
23	37	Asian	Male	Not working	Yes	17	17	0	6	5	– –
24	65	White Cypriot	Male	Part Time	No	14	11	-3	3	3	00
25	42	White British	Male	Full Time	No	2	0	-2	2	7	+-
26	20	White British	Male	Not working	No	14	19	5	7	6	00
27	48	White British	Female	Not working	No	7	8	1	3	3	++
28	40	White British	Female	Full Time	No	7	0	-7	2	2	00
29	48	White British	Female	Part Time	No	12	10	-2	4	4	– –
30	59	White British	Female	Not working	Yes	19	20	1	4	4	00
31	74	White British	Female	Retired	No	12	14	2	4	4	++
32	45	Black British ^*†*^	Female	Unassigned	Yes	12	10	-2	3	3	00
33	63	White British	Female	Retired	No	6	4	-2	3	4	00
34	64	White British	Female	Retired	No	11	9	-2	5	5	– –
35	31	Mixed ^*‡*^	Male	Full Time	Yes	14	6	-8	2	2	00

*Concordance rule details:* No change = within 1 points of 0; Slightly <5 points in concordant direction (*i.e* >0); Much >= 5 points in concordant direction; Character 1 = LBP, Character 2 = tasks; - = TQ less optimistic than RMDQ change score; + = TQ more optimistic than RMDQ change score; ND = No Data/datum. For example, a participant with a discordance status of ++ indicates a more optimistic response to both transition questions relative to the RMDQ change score. A participant with status 0- would be concordant with respect to the TQ that is worded in terms of LBP, but would have a less optimistic daily task TQ as compared to the RMDQ change score. *TQ anchors* 1=Completely better; 2=Much better; 3=Slightly better; 4=No change, 5=Slightly worse, 6=Much worse; 7=Vastly worse. ^∗^Asian or Asian British Indian. ^*†*^Black or Black British African. ^*‡*^White and Afro-American.

Informed by the baseline and four-month follow-up data, we purposively sampled participants by gender, age, employment status, and ‘discordance status’. We aimed to sample between 35 and 40 participants, within which range we expected to be approaching data saturation. We purposively sampled one participant who had missing TQ data, in order to explore the reason for non-response. We categorised discordance status both by its presence or absence, and by its direction. We defined discordance, *a priori*, as either a change on the RMDQ of any magnitude in a contradictory direction with respect to any TQ response that was not ‘no change’, or where there was a ≥ 5-point change in RMDQ score in the same direction when the response to the TQ was slightly improved (*i.e.* - status) or slightly worse (*i.e.* + status), or where there was a <=5 point change it the TQ response was ‘much improved’, unless the follow-up score was zero and thus prevented from exceeding the threshold by a floor effect. There is some consensus that a 5-point change on the RMDQ is an appropriate threshold to judge an individual responder [14]. We then coded cases of positive discordance (+) as when the *TQ response indicated a more optimistic view of recovery than the corresponding change in RMDQ score*, and negative discordance (-) when the *TQ presented a more negative response than the RMDQ score*. Concordance (*i.e* the absence of discordance, where the response to the TQ was consistent with the RMDQ) was coded as ‘0’. Since there were two transition questions we use two characters for notation. The first corresponds to the LBP TQ, which participants were asked first, and the second corresponds to the daily tasks TQ, which participants were asked second. We did not explore in the study the effect of changing the order of the transition questions.

We aimed to interview participants within four-weeks of receiving their four-month follow-up data, to minimise difficulty with recall. Interviews were semi-structured and performed either at the participant’s home, or at Warwick Medical School; whichever the participant preferred. During each one-hour interview, in accordance with a topic guide [see Additional file 1] participants were invited to describe their back pain and its impact, before being asked to review their responses to the RMDQ and TQs, and to describe their thought patterns and approach to answering the questions. If discordance was present, the reasons for it were explored.

All interviews were audio recorded and transcribed verbatim. NVivo, version 10 (QSR International, Queensland, Australia) was used to store the transcripts and facilitate data management. Coding was undertaken by DE with RF providing independent quality checks on 20% of transcripts early in the coding process. Of these, half were randomly selected and half were purposively selected by DE as those that were judged as most difficult to code. We adopted a thematic approach for analysis, coding according to a framework that was developed from initial readings of the transcripts to model data relating to thoughts and thought processes and we examined these relative to discordance between the RMDQ change score and the TQs [15]. Additional codes were added as themes emerged from the data. DE and RF developed the initial framework from coding several transcripts. Coding discrepancies were discussed and coding definitions refined. DE, RF, SP, and MU discussed the final framework and its themes and definitions, and in a research meeting the framework coding was compared to individual transcripts for triangulation. We focused on exploring themes that emerged when the participants were questioned about how they came to a decision to answer the RMDQ or TQs. We then examined these themes as a function of the participant’s coded discordance status (*vide ibid*). We explored associations by comparing data coded under different themes with characteristics and other factors. For example, associations between discordance status coding and different coded categories of interview responses were explored by forming matrices and exploring data within and between cases. Quotations were presented as exemplars of themes. Each presented quote is coded using the following syntax: [ID number, gender (m/f), age in years, discordance status (*e.g.* ++, – –, -0)].

## Results

We completed 35 interviews before approaching data saturation. Table 1 summarises the characteristics of the sample as well as the participants’ responses to the TQs and their RMDQ scores. Twenty-seven participants were female (mean age 56 (SD 12.39) years) and eight were male (mean age 45 (SD 15.32 years). Most were British (29/35). Nine worked full-time, six worked part-time, 11 were retired, six were not working, and three did not provide their work status. Within the purposive sample, the mean RMDQ at baseline was 11.6 (SD=4.39) and the four-month follow-up it was 9.0 (SD=5.89).

All participants had responded to the RMDQ at baseline. The participant with missing TQ data had simply not seen the page containing these questions. At follow-up, 15 participants were discordant with the LBP TQ and 15 were discordant with the daily tasks TQ. Twelve were discordant on both questions. In five, the TQs suggested better outcomes than the RMDQ change scores (++) and in seven it suggested worse outcomes than the RMDQ (– –). Of the heterogeneously discordant responses, two were discordant with the pain question but not the tasks, two were discordant with tasks but not pain, and one was discordant with both but in opposite directions. Seventeen participants were not discordant with either TQ.

### Roland Morris disability questionnaire

We identified two themes in terms of participants’ thought patterns that were associated with completion of the RMDQ.

#### Binary opposition thought-process

Participants’ thought-processes follow directly from the structure of the questionnaire. Participants tended to take a uniform approach involving attending to each of the RMDQ statements in turn, as they are presented, and considering each relatively objectively and dichotomously in terms of assessing whether the statement was true or not, as required by the questionnaire. For example, if a participant was considering the statement ‘I need to use a handrail when climbing the stairs because of my back’, this was something that they judged that either they needed to do, or that they did not need to do; *i.e.* a binary opposition. The statements did not in general lead to debate, difficulty, or dilemma, but directly to a decision and concluding statement of agreement or disagreement. No associations emerged between gender, age, employment status, or discordance status.

*“That one I do* [change positions frequently because of back pain]*, you know, if I’m sitting on the sofa watching the TV, I tend to move and then move to the other side and, you know, put my feet up. I do still do that.*” - (Participant 2, 70 yo White British Female, ++)

*“No, I don’t stay at home most of the time. Do I change positions frequently? No. Walk more slowly? Don’t think so. Not doing jobs I usually do around the house? I have to take some care cleaning the bath, but it’s only that.... Handrail? No*. ” - (Participant 12, 64 yo White British Female, -0)

#### Temporal irrelevance

We identified the second theme from several participants who commented that the RMDQ wording asks about ability ‘today’. Participants felt this risked responses being over-influenced by an atypically ‘good’, or an atypically ‘bad’ day, and thus the RMDQ failed to capture relevant information about symptoms over time.

“On this day is it bad, you know?... But if it’s on a good day, then it’s OK, but like I know mine obviously did go really quite high then... that particular week, I’d been sitting a lot because I’d been out for meals, as I say and sort of down to K*** as well, which made my problem worse then in that week.... possibly within [the RMDQ should inquire about] *the last month, because that’s the thing, it isn’t always just that day, is it? It depends on what’s made it worse today than last week sort of thing.”* - (Participant 21, 65 yo White British Female, – –)

*“... it does say today, doesn’t it?... I was taking it more as a general... I mean, today is underlined as well.... there are things... like this one, I could say I’ve done this more as a general one and that one more as, er.. how I was on that day*.” - (Participant 24, 65 yo White Cypriot Male, 00)

*“... it would be good if you did something like every couple of days; instead of just doing it the once, to do it over a week period or something?... Because sometimes you can be really good and you fill it in and you think, oh, yeah, I can do that, I can do that! But then the next day, I couldn’t!”* - (Participant 28, 40 yo White British Female, 00)

### Health transition question (TQ)

**Figure 1:**
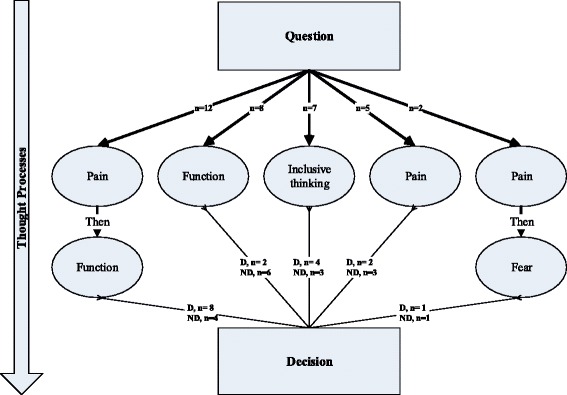
Decision-makingpathways. The figure shows the five emerging themes in descriptions of thought pathways, and the association with discordance status. D=discordant; ND=not discordant.

Five distinctive thought pathways emerged as themes in the data that related to thought processes during participants’ responses to TQs; particularly in terms of the prioritising and order of consideration of health domains (Figure 1). Participants either thought about pain then function; function then pain; their willingness to globally accept their health state globally; pain alone; or pain and then fear. The pathways are listed in order of the prevalence expressed within our purposive sample. Notwithstanding the differing categories and chronological orders of thought processes observed, we note that generally thoughts about the pain were at the forefront of thought processes. The thought process for some was simple and generally required little mental debate; however, most made a more considered appraisal. This framework accommodates all of the qualitative data relating to thoughts and thought pathways.

The following sections summarise each of the thought pathways identified within the framework. They include relevant example quotes that were typical of responses, and describe any discordances observed and their directions.

#### Participants who thought about their pain and then their function

Of the 12 participants who thought about pain first and then function [ID= 1, 8, 9, 16, 19, 22, 23, 25, 29, 31, 33, 34], eight had discordant RMDQ and TQ scores. Of these, seven had less optimistic responses (*i.e.* were negatively discordant) on either LBP or daily task variants of the TQ and four had less optimistic responses on both (*i.e* four had a discordance value of – –). These four responses may be qualitatively different in that these participants expressed more negatively emotional responses than those who were classified as being positively discordant and who expressed more optimism. Thoughts about function tended to be centred around what participants could or could not do (*e.g.* in terms of work or leisure).

*“Yeah, because if the pain is there you can’t do things, can you?”* - (Participant 19, 73 yo White British Female, 00)

*“I think how much does it hurt, I suppose... Yeah. As I say, if we walk for a long distance, that definitely starts aching.”* - (Participant 8, 74 yo White British Female, 00)

*“...But I think, yeah, the pain, the level of pain, the intensity, is what determines everything else in your life; whether it’s getting out of a chair, whether you need help getting dressed. I mean, there were occasions when I did need help to get dressed and so, therefore, if you say what is it that makes you feel better, it is in some way either lessening or losing the pain, because obviously that makes life easy then.”* - (Participant 1, 61 yo White British F, – –)

*“Well, yeah, you see, when I’m sitting like this, I can feel a bit of tenderness sort of thing and I suppose it’s until you come to walk, and you think, well, say, tomorrow I might have a bit of pain, but I don’t have the pain every day. And this is probably where you think, you think it’s going but it never goes.”* - (Participant 31, 74 yo White British F, ++)

#### Participants who thought first about function

Of the eight participants who thought first about function [ID= 5, 6, 11, 17, 18, 28, 32, 35], six were not discordant (*i.e.* had a discordance value of 00) and two were discordant. Of those who were discordant, there was no notable directional association.

*“ I could actually fit more into the day... Housework was easier... The washing up would normally take you five minutes, but it took, say, half an hour, so it was the time thing of getting things done, you could do more. Basic things like shopping was easier.”* - (Participant 18, 56 yo White British Female, 00)

*“It would be* [better] *from the first time that it happened, you know, about getting in the car and, you know, sitting for long periods. Because sometimes I do have to sit at the computer for quite a while and whatever, and I would say it was slightly better now, because I can sit longer and I can do a little bit more; whereas before when it first happened, I would say no”.* - (Participant 5, 45 yo White British Female, – –)

*“... How far I can walk... How long I can stand for; those kind of things.”* - (Participant 32, 45 yo Black or Black British African Female, 00)

#### Participants who used inclusive thinking

Seven participants thought about their response in relatively more global or complex terms [ID=2, 3, 4, 14, 15, 24, 27]. Three were concordant and four were discordant, with three having more optimistic responses to the TQ than indicated by the RMDQ change score (*i.e*. were positively discordant) and one had less optimistic TQ responses than indicated by the RMDQ change score (*i.e.* were negatively discordant) for both variants of the TQ. In this category, thinking was principally a global appraisal, for example, incorporating multiple factors and possibly interactions between those factors, or an overview of a situation, rather than immediately thinking of pain or another discrete factor. We have labelled this ‘inclusive thinking’. Responses reflect an adjustment or an additional consideration that is incorporated in the response to the TQ that is independent from responses to the RMDQ. There may have been some suggestion that those who were positively discordant had exceeded their global expectations.

*“...But, you know, at the end of the day, I have to accept that I’m older now and there’s general wear and tear in my body; to me there’s no doubt about that. So I don’t think I’m ever going to get back to how I was ten years ago, and I think this is something I’m going to have to live with, you know? So is my ability slightly better?”* - (Participant 2, 70 yo White British Female, ++)

*“I put that because when I’m not at work and I’m not under pressure at work, I feel slightly better because I can take my time doing the normal daily things that, if you’re in a work pattern, that you would do automatically.”* - (Participant 3, 57 yo White British Female 00)

*“Well, I don’t personally think I’ll ever be ever without back pain. There will be days and weeks that I’ll have it, so it’s never going to be... it could maybe reach much better, but it will never be completely better; you’ll never get to that, only much better.”* - (Participant 4,61 yo White British F, – –)

*“There’s a lot of residual effect of back pain that it does restrict the things that you do; although I try not to let it restrict the things that I want to do. Up until last year, we were skiing every year and it didn’t stop me skiing. I found the more exercise I did, good exercise, the better off my back was, because I became fitter and my back was stronger because of it... ”* - (Participant 14, 54 yo White British Male ++)

*“You know you’ve still got it there, you know it’s still not 100% and for my case, it will never be 100%, but you just know that if you do what you should do and how you go about it, you’ll be fine.”* - (Participant 15, 55 yo White British Male 00)

*“In my head I felt slightly better. That may have been a day when I was feeling a little bit more positive; even though I was in pain, maybe part of me was thinking, oh, this could be something, it could get better.”* - (Participant 27, 48 yo White British Female, + +)

#### Participants who thought only about their pain

Of the five participants who thought only about pain when responding to the TQ [ID=7, 10, 20, 21, 26], two were discordant and three were not discordant. One had more optimistic responses to the TQ than indicated by the RMDQ change score (*i.e.* was positively discordant) for both LBP and daily task variants, and one had less optimistic responses to the TQ than indicated by the RMDQ change score (*i.e.* was negatively discordant) for both variants.

There was some suggestion that for the participants in this pathway the decision-making process was more clear-cut and without lengthy mental debate. There were no notable associations with discordance in either direction.

*“My pain. Has anything changed? No, it’s still the same. My pain is still the same. It’s simple!”* - (Participant 7, 23 yo Asian or Asian British Female, 00)

*“I think I thought am I in more pain now than I was when I started?... I think that’s what I’ve based it on, whether my pain was any better or worse from start to finish and it gradually got worse.”* - (Participant 10, 57 yo White British Female 00)

*“Yeah... well, no, just the pain, when I’m just having it bad... Yeah, this is just about the pain. If I was to say with the mental stuff and not being able to do anything, then it would be that one that’s really worse.”* - (Participant 26, 20yo White British Male, 00)

*“Well, because I still have back pain.”* - (Participant 21, 65 yo White British Female, – –)

#### Participants who thought about their pain and then fear

Finally, of the two participants who thought about pain and then fear [ID=12, 30] (that is, in terms of being worried about doing further damage to their back), one was concordant and one had less optimistic TQ responses than indicated by the RMDQ change score (*i.e.* was negatively discordant) on only the LBP TQ (*i.e.* a discordance value of –0)

*“... it’s obviously not going to be there 24/7, it’s going to ease off. But when you’re getting it, you don’t think it’s going to ease off; you think, oh, and you get this fear that something is going to happen to you, I keep thinking... kept thinking, will I end up on a stretcher and end up where I’m paralysed, because it’s been so extreme!”* - (Participant 30, 59 yo White British Female 00)

*“When I say clicking, it doesn’t nearly describe it. You’d be walking along normally, normally, normally, and then suddenly, quiet unpredictably, get this really sharp pain as though something is going to break!... Yeah, because what (this) was worrying me, frightening me and sent me to the doctor... was, oh, I’m doing some damage by walking; if I carry on with this, I’m not going to be able to walk!”* - (Patient 12, 64 yo White British Female, -0)

### Summary of discordance patterns within the framework

There were more participants who thought about pain and then function than in any other of the five categories. They were more likely to have RMDQ scores that were discordant with TQ scores and the majority had higher RMDQ change scores. In those who thought about pain only, TQ responses largely matched RMDQ responses. In the inclusive thinking category, there is some indication that those who had more optimistic TQ scores than indicated by lower RMDQ change score had in some way exceeded their own *a priori* expectations for their improvement.

## Discussion

The results of this study show how it is possible that patients can say they have improved or deteriorated whilst having a contradictory RMDQ change score: for some patients the assessments are in different domains. Ability to perform daily tasks (*i.e.* the domain of the RMDQ) can be independent of back pain. Whilst participants who thought principally about their function when answering TQs tended to have concordant TQ and RMDQ responses, consideration of pain before function, the predominant pathway, was associated with having a less optimistic TQ response than RMDQ score, suggesting that pain is the primary driver of the response to the daily tasks TQ as well as for the LBP TQ.

This finding is problematic since it suggests that some people do not attend to thinking about their back pain when completing the RMDQ. This may render the RMDQ unsuitable for use as a primary outcome measure in back pain trials if the objective is to determine individual change, or between-group differences in back pain. If the focus were on improving daily living, it may provide useful and relevant information.

In 2014, the National Institutes of Health (NIH) task force recommended using Patient Reported Outcomes Measurement Information System (PROMIS) measures as a minimum dataset in all NIH-funded LBP research, recommending that the RMDQ could be a substitution for the PROMIS physical activity items if more extensive legacy measures are required [16]. The RMDQ may be well-placed for specifically measuring function. Notwithstanding our findings, we note that the RMDQ usually shows to be the superior instrument, strictly in terms of its clinimetric performance when compared to other back-specific measurement instruments; it has convincingly been shown to be reliable and responsive [6,10,17-19].

Hush *et al.* have highlighted that participants have expressed concern that the RMDQ had not seemed relevant to them and that the time-frame of assessment of the RMDQ was thought to be problematic [20]. Our findings also suggest that some participants have concerns surrounding relevance. It was felt that specific weaknesses of the RMDQ included its focus on the day of completion and its failure to measure recent recollection of ability, confirming the original report of this issue. From a clinical standpoint this could be considered a relative weakness over measures that have a temporal component because the trajectory of back pain from day-to-day is known to be erratic [3].

Hush *et al.*, in 2012, reviewed patients’ views on recovery from low back pain [21]. This, combined with a review of recovery measurement over the past decade, [22] informed a workshop in which expert opinion was sought on standardised recovery measures using the nominal group method. For measuring recovery, they recommended the Global Back Pain Recovery Scale – a transition question worded in terms of recovery – and the Patient Generated Index (PGI) of Life-Back Pain [23,24].

Arguably a distinction should be made between recovery measures and outcome measures. Kamper *et al.* note the absence of a definition of recovery, which we suggest inherently relates to the individual patient [22]. Measurement of recovery and the analysis of the number of recoveries in trials is emphatically useful, since it facilitates interpretation of trial outcomes [25,26]. Outcome measurement should be considered more general; inasmuch as it charts the change in a latent variable, on aggregate, regardless of whether recoveries (or deteriorations) have occurred in individual patients [27]. The minimally important between-group (population-level) difference of course usually forms the basis of the sample size calculation for trials, and since magnitudes of importance at the population-level can differ from those at the individual level, it is important to separate the level of interest [28].

In a systematic review, in 2014, of qualitative research on the impact of back pain on patients’ lives, Froud *et al.* highlighted a discord with domain coverage of outcome measures recommended in core sets [29]. As the Patient Generated Index (PGI) permits participants to define what matters most to them and then rate the change in those domains, its use would improve the relevance of outcome measurement in trials generally as well as for measuring recovery in individuals [21,22]. However, there may be an inherent clinimetric weaknesses in the current design of the PGI. Participants are asked to both weight and rank their nominated domains of measurement. This adds an additional source of variance, which can disadvantage its metric performance relative to other instruments. For example, when exploring reliability, the increased within-person variance term is bound to (reasonably assuming it to be greater than zero) attenuate the coefficient that is often used to summarise the instrument’s reliability/agreement, [30,31] and could render the PGI less attractive from a clinimetric perspective, relative to other available instruments. We would encourage clinimetric comparisons of variations on PGI designs; for example, by removing the weighting, or permitting a rating only at baseline.

The validity of using TQs in general has been questioned. Guyatt *et al.* [9] suggest that correlations of less than 0.5 between the change in PROM score and TQ should be grounds for doubting the construct validity of the TQ. Indeed, criticisms of using TQs centre on the rating’s likelihood to be more correlated with the follow-up health state and PROM score, than baseline state and PROM score, essentially highlighting that respondents may not correctly recall their baseline health state. The criticism may underline another more fundamental question surrounding how interested we should be in the ability of the TQ to measure change. Guyatt also points out that if the TQ measured change rather than being driven by current health state, then one would expect to find a correlation between baseline PROM score and the TQ, and follow-up PROM score and the TQ that is present, equal, and opposite [9]. In addition, in a linear regression model the follow-up PROM score should explain a significant and material proportion of the variance in the TQ, which is often not the case [8,9,32]. However, we consider that PROM scores are most useful in pragmatic clinical trials, where establishing the effectiveness rather than the efficacy of an intervention is the primary objective [33]. In pragmatic trials the focus on the follow-up health state is sensible since pragmatic trials are chiefly done to inform policy and in the case of back pain where we are concerned with morbidity, rather than mortality, the objective and post-treatment view of the patient is more valuable to decision-making and directing health spends. We agree with Ostelo *et al.* that most physicians would be reluctant to label a patient as improved or deteriorated against that patient’s personal assessment [34]. We support the recommendation of Hush *et al.* of using the TQ to determine when recovery has occurred, and suggest that it might also be useful as an outcome measure when the focus is on health transition at the population-level. Hush *et al.* [21] recommend using an 11-point outcome measure based on a review by Kamper *et al.* and Preston and Coleman’s work on optimal category scales, in 2000 [23,35].

Lauridsen *et al.*, in 2007, compared a 7-point TQ with a 15-point TQ for use as an external criterion for estimating MIC on PROM instruments, within a group of 181 low back or leg pain patients receiving best care who had completed five validated PROMs [36]. They also examined different stringencies. They observed no discriminatory difference, but as the 7-point scale produced a slightly more conservative estimate they recommended the 7-point scale for use as an external criterion.

Whilst potentially useful as an outcome measure at population-level, as well as to measure recovery at the individual-level, we would caution against using a TQ as an external criterion to estimate RMDQ MIC thresholds on ROC curves [37]. To be suitable for this purpose, the TQ would need to a useful proxy measure of change, and an accurate proxy measure of change within the same domain as the RMDQ. On both counts the TQ is inadequate; correlations and regression modelling from other studies show that the TQ does not measure change, [8,9,32] and the current study suggests that regardless of TQ wording, it is pain that drives the transition question and that this is independent of the domain measured by the RMDQ.

We noted that we stopped the research when we were approaching data saturation. We prefer the term ‘approaching data saturation’ over ‘data saturation’, which we suggest may be a slightly unfortunate term. Whilst often used to describe the point when no novel themes are emerging, it may be an unrealistic or inaccurate descriptor in that it is actually only the incidence of novel themes decreases with data acquisition. Diminishing returns and practicalities mean that when data saturation is being approached it is reasonable to stop the research. With a larger sample, we may have been able to obtain more novel data. However, after 35 interviews in our study novel themes were diminishing, and the willing and eligible persons remaining had characteristics that had already been well-sampled; as such we recognise a limitation. However, it is a limitation that we suggest applies to most qualitative research. The completion of 35 interviews exceeds the size of many qualitative studies yet the sample was not so large as to limit our capacity to analyse data [38]. Results of qualitative research should not be considered generalisable, due to purposive sampling, and in contrast to representative sampling required for inference; however, our results are transferable, insofar as they should reflect the range of themes from the population.

Our results give cause for concern surrounding the use of the most common primary outcome measure in back pain trials; the RMDQ [10,39]. We have supported suggestions for exploring the use of the TQ and PGI as a primary outcome measure, for their relevance to patients. However, we would not seek to discourage the development of new instruments, especially those which exploit modern developmental approaches, and involve patients in the development. One hazard in reconsidering primary outcome measure use in LBP trials, could be that lessening RMDQ use may pave the way for a more heterogeneous usage of outcome measures; the very scenario that the influential recommendations for core-sets in 1998 and 2000 was originally intended to correct [40,41]. Kamper *et al.* have shown that between 1999 and 2008 measurement of recovery has been diverse utilising a vast array of different instruments and approaches [22]. We are currently exploring trends in outcome measurement, and measurement heterogeneity, in back pain trials over the past three decades (Froud R, et al. A systematic review of outcome measure use and reporting methods in low back pain trial reports published between 1980 and 2011. *In preparation*.). Notwithstanding the risk of increasing heterogeneity, the current situation in which the most commonly used assessment method in trials and practice does not correspond well to perceived changes in back pain, is undesirable. One reason for heterogeneity may be the absence of a compelling primary outcome measure, making the argument for not developing new instruments on the grounds of increasing heterogeneity difficult to uphold. Given the huge costs and burden of back pain on society, it could be viewed as regrettable (possibly even unethical) that many millions are spent each year on assessing health technologies for the improvement of back pain, when one of the most commonly used back pain outcome measures may not be capturing what is relevant to patients.

At the design stage of any new instrument, we suggest that researchers might consider whether there are any potentially useful viewpoints that have hitherto been neglected, for example, by including linguists, psychologists, psycho/clinimetricians and sociologists in addition to patients and clinicians. We support ongoing work aimed at reconsideration of these core sets, with groups aligned with the Core Outcome Measures for Effectiveness Trials (COMET) initiative, but emphasise the importance of evaluating the design and clinimetric performance before recommending the inclusion of an instrument in core-sets. In assessing design and performance of instruments, the COnsensus-based Standards for the selection of health Measurement INstruments (COSMIN) check-list may be useful. The aim of the COSMIN initiative is to improve the selection of health measurement instruments (www.cosmin.nl). Following a Delphi study, the group developed a critical appraisal tool and standards for evaluating the methodological quality of studies on the measurement properties of health measurement instruments.

If new candidate(s) are selected as a preferred primary outcome measure for use in low back pain trials, a smooth transition may need to be managed. With the life of a clinical trial spanning upwards of five-years from conception to publication, some degree of fragmentation in primary outcome measure use may be unavoidable and should be considered as it would need to be clear whether or not there would be sufficient buy-in from trialists to minimise fragmentation. The Delphi method with a large panel of trialists may be useful in this regard to see beforehand whether consensus on change can be achieved; it is not clear that recommendations on outcome measurement and core sets are having any impact on altering practice ([39], Froud R, et al. A systematic review of outcome measure use and reporting methods in low back pain trial reports published between 1980 and 2011. *In preparation*). There may also be a detrimental effect to comparisons between trials; although additionally standard effect sizes and responder analyses would go a long way to mitigate this [16,25].

## Conclusions

Approaches to primary outcome assessment in back pain needs re-assessment. People do not think about their back pain when they complete the most commonly used primary outcome in back pain trials–the RMDQ. Researching a more relevant substitute instrument for use as a primary outcome measure in back pain trials needs further consideration as do transition strategies and ways to improve trialist buy-in. TQs should not be used to anchor RMDQ MIC thresholds as these may not provide a valid proxy of change in the latent construct measured by the RMDQ but are primarily driven by pain.

## Additional file

Additional file 1
**Topic Guide.** A PDF (.pdf) file showing the original Topic Guide as an indication of the topics covered in our interviews. We note that items 1, 2 and 4 are additional areas that were explored for a linked study. The document provided is the initial topic guide as at the start of the study and is provided as an indication only, as questions prompts and probes evolve with interviews.

## Abbreviations

COMET: Core outcome measures for effectiveness trials
COSMIN: Consensus-based standards for the selection of health measurement instruments
LBP: Low back pain
MIC: Minimally important change (individual level)
NHS: National Health Service
NIH: National Institutes of Health
NRES: National Research Ethics Service
PROMs: Patient reported outcome measures
REC: Research Ethics Committee
RMDQ: Roland Morris Disability Questionnaire
SD: Standard deviation
TQ: Transition question
UK: Unlited Kingdom
YLDs: Years lived with disability
