# Supplementary material for: Primary outcome measure use in back pain trials may need radical reassessment
Source: BMC Musculoskelet Disord. 2015 Apr 14;16:88. doi: 10.1186/s12891-015-0534-1 (PMC4419506; doi:10.1186/s12891-015-0534-1)
Supplement: Additional file 1 — Topic Guide. A PDF (.pdf) file showing the original Topic Guide as an indication of the topics covered in our interviews. We note that items 1, 2 and 4 are additional areas that were explored for a linked study. The document provided is the initial topic guide as at the start of the study and is provided as an indication only, as questions prompts and probes evolve with interviews. [file 12891_2015_534_MOESM1_ESM.pdf]

Additional interview study: Exploring Perceptions of Important Change (EPIC)

INTERVIEW TOPIC GUIDE (for semi-structured interview)

NOTE: ITEMS 1, 2 AND 4 ARE ADDITIONAL AREAS THAT WERE EXPLORED FOR A LINKED STUDY. THE DOCUMENT PROVIDED IS THE INITIAL TOPIC GUIDE AS AT THE START OF THE STUDY AND IS PROVIDED AS AN INDICATION ONLY, AS QUESTIONS PROMPTS AND PROBES EVOLVE WITH INTERVIEWS.

Introduction

Thank you for agreeing to see me today. I really appreciate your giving up your time and being willing to share your thoughts about your back pain. As you're probably aware, we plan to use the information you give us to help improve the ways we evaluate treatments for back pain. I mentioned that I'd like to record the interview so that I can pay attention to what you're telling me, but I might also make some notes to help me keep track of my questions and remind me to pick up on something that you've said – is that OK? Some of my questions might seem to pry into personal areas – I know some people find some things related to their back pain difficult to talk about or embarrassing, so please do let me know if you want a break – and of course you are free to finish the interview at any time. Do you have any questions before I start? So, I'm going to turn on the tape recorder now, if that's OK?

1. History and nature of the back pain

First of all, I'd like you to tell me a bit about your back pain.
What impact you feel your back pain has had on your life?

Prompts and probes:

- *When did you first start to have back pain?*
  - *Chronology, continuous or episodic, nature of pain over time?*
    - *Change in intensity, impact and management over time?*
- *Are there any activities or movements that particularly make your back worse?*
  - *Are there things you don't do, or used to avoid, through fear of making it worse?*
- *Anything that you've found you can/could do to make it better?*
- *What treatments have you had for your back pain?*
- *How has having low back pain affected your life? What parts of your life were affected most?*
  - *How has your back pain changed over time?*
- *Can you think of a fairly recent time when the pain was particularly bad? At that time, how bad was your pain?*
  - *At that time, what did you think the pain might mean?*

- *What are/were your expectations with regards to your back pain resolving?*
- *Have they changed over time? (probing response-shift)*
- *Sometimes when people are in severe pain, it changes the way they think about themselves. Does/did that ever happen to you? If so, can you describe the change? Do/did you have a mental image of yourself, which you could share with me?*

2. Changes in back pain since beginning treatment in IMPACT

What lead to you taking part in the IMPACT trial?

Prompts and probes:
Previous research and treatment experience
Opportunity for different/new treatment you wouldn't otherwise have received?
Please tell me a little about your experience of being part of the study
Personal experience of treatment
Thoughts about being 'measured'
Choice and control over treatment (because the literature seems to suggest that sense of control influences experiential outcomes)

How has your back pain changed since you began treatment on the IMPACT trial?

Prompts and probes:
- *For example, has it go better, worse, or stayed the same?*
    - *How have the changes affected your life? What has been the impact of the changes to your life?*
- *Has the intensity, or nature of the pain changed over time?*
    - *How has your attitude towards your back pain changed?*
    - *How have your feelings toward your back pain changed over time? Probe activities, social & functional.*
- *What did you think of the treatment you received?*
    - *How helpful did you find it? Was there any aspect of treatment you found particularly helpful?*

3. Whether changes were captured by the outcome measures used

Completed questionnaires will be available

What did you think of the questionnaires (these will be shown to refresh the participant's memory) you completed before treatment and then three months later, just recently?

Prompts and probes:
- *How were the questions relevant to you?*
- *Did the questionnaires cover everything you feel was important in relation to your back pain?*
    - *Any questionnaires seem more relevant/acceptable than others?*
        - *In what ways?*

- *How much did you think about each question?*
    - *Did the questionnaires hold your concentration?*
        - *If no – why do you think they lost your interest? (e.g. too long, too short, or irrelevant?)*
- *What would you add to the list of questions?*
    - *Probe why, what makes the suggested questions more relevant?*
- *What items do you find to be the least relevant/important?*
- *You mentioned that you enjoy <specific activity> – so I wonder whether the statement (e.g) 'I walk more slowly than usual because of my back' one that you feel has more importance for you than some of the others? (i.e. if not already explored)*

Thought processes

I'd like to have a look at some of your responses to the questionnaires with you and discuss why you answered in the way that you did.

Prompts and probes:

- *What kind of day were you having when you filled them in? How were you feeling -*
    - *Generally, and with respect to your back pain?*
    - *If you'd answered the day before or the day after would anything have been different?*
- *What were you thinking about when you answered this (the health transition question) question? What did it mean to you?*
- *What did you think about when you read the statement 'I stay at home most of the time because of my back'?*

Probe comparisons – e.g. time when you had no back pain

One question you answered <interviewer shows question> was simply 'Since beginning treatment, has your back pain:

1. completely recovered, 2. much improved, 3. slightly improved, 4. no change, 5. slightly worsened, 6. much worsened, and 7. vastly worsened.?'

I wonder how you came to score this the way you did – what did you consider?

- What was more or less important?
- How did you decide between the answers?

Comparison between single question and change in questionnaire score

There will be conditional lines of questioning in this section. Participants will only be asked those questions that are relevant to them. If their questionnaire scores and the transition question agree, *i.e.* both suggest either an improvement, no change, or a deterioration, then whether or not the participant gave the same considerations to both outcome measures will be explored. If they are discordant, *i.e.* answering much improved to the single question, but questionnaire score(s) indicate deterioration, then reasons for the differences will be explored.

The following questions are listed under possible outcomes from the single question, but comparisons to the participants questionnaire score(s) will be made throughout.
If answer to single question is ‘worse(ned)’:

In what way was your back pain worse?

Prompts and probes:

- *In what way do/did you feel your back pain had worsened?*
- *What specific aspects relating to your back pain do you think have deteriorated?*
- *When you think about how you pain has gotten worse, what were the most important changes?*
- *Do you think the questionnaires captured that?* (Especially important to probe if change in questionnaire score indicated improvement)
- *Even though generally it has worsened, is there any aspect of your back pain that has improved?*

If answer to single question is ‘unchanged’:

Can you recall what important aspects about your back pain were unchanged when you answered this?

Prompts and probes:

- Although it was unchanged overall, was there anything that had changed?
- *Do you think the questionnaires captured that?*
- *Are there any aspect of your back pain that has improved/worsened?*
  - *Probe whether questionnaire and single question captured these*

If answer to single question is ‘improved’:

What do you think it was that made it seem better overall?

Prompts and probes:

- *In what way do/did you feel your back pain improved?*
- *When you think about how you pain improved, what were the most important changes?*
- *Do you think the questionnaire captured that?*
  - (Especially important to probe if change in questionnaire score indicated worsening)
- *What aspects of your function do you think has deteriorated?*
- *Even though generally it has improved, is there any aspect of your back pain that has worsened?*

4. Thoughts on how the outcome questionnaires could be improved

How do you think we could better capture the changes in your back pain and the impact it has on your life?

Prompts and probes:

- *What should we have asked?*
- *Was there anything that you weren't asked about but that you would like to have been asked about?*
- *Do you think there was anything in the questionnaire that shouldn't have been in there?*
- *How comfortable were you when completing the questions?*
- If we could ask only one question, which would it be?

5. Closing the interview

Is there anything else that you would like to tell me about?
Thank you very much indeed for your time – your responses to my questions have been extremely valuable.
